# *N*,*N*′-Bis(3-phenyl­allyl­idene)biphenyl-2,2′-diamine

**DOI:** 10.1107/S1600536809000804

**Published:** 2009-01-14

**Authors:** Saeed Dehghanpour, Farzaneh Afshariazar, Shan Gao, Seik Weng Ng

**Affiliations:** a Department of Chemistry, Alzahra University, Vanak, Tehran, Iran; b School of Chemistry and Materials Science, Heilongjiang University, Harbin 150080, People’s Republic of China; c Department of Chemistry, University of Malaya, 50603 Kuala Lumpur, Malaysia

## Abstract

In the title Schiff base, C_30_H_24_N_2_, the complete molecule is generated by a crystallographic twofold axis; the aromatic rings of the biphenyl unit are twisted by 60.78 (1)°. The imine double bond has a *trans* configuration.

## Related literature

For a list of the crystal structures of Schiff bases formed by condensing biphenyl-2,2′-diamine with aldehydes or ketones, see: Dehghanpour *et al.* (2009 ▶). 
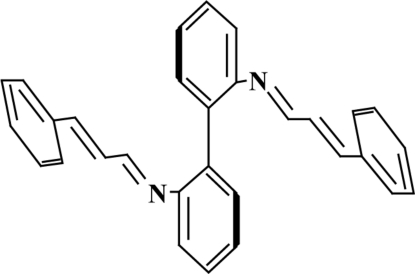


## Experimental

### 

#### Crystal data

C_30_H_24_N_2_
*M* *_r_* = 412.51
Orthorhombic, 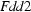

*a* = 15.4354 (12) Å
*b* = 31.783 (2) Å
*c* = 9.6188 (8) Å
*V* = 4718.8 (6) Å^3^
*Z* = 8
Mo *K*α radiation
μ = 0.07 mm^−1^
*T* = 295 (2) K
0.27 × 0.21 × 0.16 mm

#### Data collection

Rigaku R-AXIS RAPID diffractometer
Absorption correction: multi-scan (*ABSCOR*; Higashi, 1995 ▶) *T*_min_ = 0.982, *T*_max_ = 0.989
11331 measured reflections
1427 independent reflections
1021 reflections with *I* > 2σ(*I*)
*R*_int_ = 0.029

#### Refinement

*R*[*F* ^2^ > 2σ(*F* ^2^)] = 0.034
*wR*(*F* ^2^) = 0.110
*S* = 1.07
1427 reflections
145 parameters
1 restraint
H-atom parameters constrained
Δρ_max_ = 0.11 e Å^−3^
Δρ_min_ = −0.15 e Å^−3^

### 

Data collection: *RAPID-AUTO* (Rigaku, 1998 ▶); cell refinement: *RAPID-AUTO*; data reduction: *CrystalStructure* (Rigaku/MSC, 2002 ▶); program(s) used to solve structure: *SHELXS97* (Sheldrick, 2008 ▶); program(s) used to refine structure: *SHELXL97* (Sheldrick, 2008 ▶); molecular graphics: *X-SEED* (Barbour, 2001 ▶); software used to prepare material for publication: *publCIF* (Westrip, 2009 ▶).

## Supplementary Material

Crystal structure: contains datablocks I, global. DOI: 10.1107/S1600536809000804/bt2848sup1.cif

Structure factors: contains datablocks I. DOI: 10.1107/S1600536809000804/bt2848Isup2.hkl

Additional supplementary materials:  crystallographic information; 3D view; checkCIF report

## Figures and Tables

**Fig. 1.**
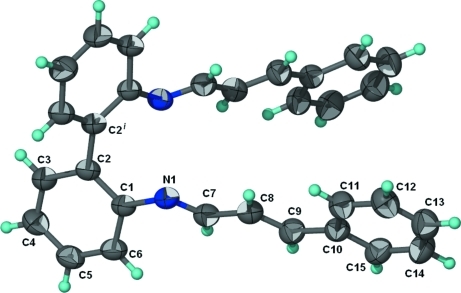
Anisotropic displacement ellipsoid plot (Barbour, 2001); displacement ellipsoids are drawn at the 50% probability level, and H atoms as spheres of arbitrary radius. (Symmetry code i: -x, -y, z).

## supplementary crystallographic information

### Experimental

Biphenyl-2,2'-diamine (5 mmol) and cinnamaldehyde (10 mmol) were dissolved in diethyl ether (50 ml). The mixture was stirred for 30 min. Evaporation of the solvent gave a solid that was recrystallized from ethanol twice. Yield: 80%. CH&N elemental analysis. Calculated for C_30_H_24_N_2_: C 87.35, H 5.86, N 6.79%; found: C 87.30, H 5.81, N 9.82%.

### Refinement

H atoms were placed in calculated positions [C—H 0.93 Å and *U*_iso_(H) 1.2*U*_eq_(C)], and were included in the refinement in the riding-model approximation. Friedel pairs were merged

### Crystal data

**Table:**

C_30_H_24_N_2_	*F*(000) = 1744
*M**_r_* = 412.51	*D*_x_ = 1.161 Mg m^−^^3^
Orthorhombic, *F**d**d*2	Mo *K*α radiation, λ = 0.71073 Å
Hall symbol: F 2 -2d	Cell parameters from 7049 reflections
*a* = 15.4354 (12) Å	θ = 3.2–27.5°
*b* = 31.783 (2) Å	µ = 0.07 mm^−^^1^
*c* = 9.6188 (8) Å	*T* = 295 K
*V* = 4718.8 (6) Å^3^	Cuboid, light yellow
*Z* = 8	0.27 × 0.21 × 0.16 mm

### Data collection

**Table:**

Rigaku R-AXIS RAPID diffractometer	1427 independent reflections
Radiation source: fine-focus sealed tube	1021 reflections with *I* > 2σ(*I*)
graphite	*R*_int_ = 0.029
Detector resolution: 10.000 pixels mm^-1^	θ_max_ = 27.5°, θ_min_ = 3.2°
ω scans	*h* = −20→19
Absorption correction: multi-scan (*ABSCOR*; Higashi, 1995)	*k* = −41→41
*T*_min_ = 0.982, *T*_max_ = 0.989	*l* = −12→12
11331 measured reflections	

### Refinement

**Table:**

Refinement on *F*^2^	Primary atom site location: structure-invariant direct methods
Least-squares matrix: full	Secondary atom site location: difference Fourier map
*R*[*F*^2^ > 2σ(*F*^2^)] = 0.034	Hydrogen site location: inferred from neighbouring sites
*wR*(*F*^2^) = 0.110	H-atom parameters constrained
*S* = 1.07	*w* = 1/[σ^2^(*F*_o_^2^) + (0.0608*P*)^2^ + 0.8672*P*] where *P* = (*F*_o_^2^ + 2*F*_c_^2^)/3
1427 reflections	(Δ/σ)_max_ = 0.001
145 parameters	Δρ_max_ = 0.11 e Å^−^^3^
1 restraint	Δρ_min_ = −0.15 e Å^−^^3^

### Special details

**Table:**

Geometry. All e.s.d.'s (except the e.s.d. in the dihedral angle between two l.s. planes) are estimated using the full covariance matrix. The cell e.s.d.'s are taken into account individually in the estimation of e.s.d.'s in distances, angles and torsion angles; correlations between e.s.d.'s in cell parameters are only used when they are defined by crystal symmetry. An approximate (isotropic) treatment of cell e.s.d.'s is used for estimating e.s.d.'s involving l.s. planes.

### Fractional atomic coordinates and isotropic or equivalent isotropic displacement parameters (Å^2^)

**Table:**

	*x*	*y*	*z*	*U*_iso_*/*U*_eq_	
C1	−0.01193 (13)	0.05064 (6)	0.5676 (2)	0.0523 (5)	
C2	−0.02328 (13)	0.02045 (6)	0.4634 (2)	0.0541 (5)	
C3	−0.07746 (15)	0.02974 (7)	0.3523 (3)	0.0641 (6)	
H3A	−0.0859	0.0097	0.2831	0.077*	
C4	−0.11943 (16)	0.06833 (8)	0.3424 (3)	0.0724 (7)	
H4A	−0.1561	0.0739	0.2679	0.087*	
C5	−0.10622 (16)	0.09796 (7)	0.4433 (3)	0.0695 (7)	
H5A	−0.1333	0.1240	0.4363	0.083*	
C6	−0.05334 (14)	0.08962 (6)	0.5546 (3)	0.0609 (6)	
H6A	−0.0449	0.1101	0.6223	0.073*	
C7	0.03355 (16)	0.05664 (7)	0.7976 (3)	0.0598 (6)	
H7A	−0.0185	0.0702	0.8165	0.072*	
C8	0.09664 (17)	0.05217 (7)	0.9066 (3)	0.0629 (6)	
H8A	0.1467	0.0370	0.8870	0.075*	
C9	0.08822 (15)	0.06818 (7)	1.0333 (3)	0.0637 (6)	
H9A	0.0374	0.0829	1.0516	0.076*	
C10	0.15066 (15)	0.06500 (7)	1.1471 (3)	0.0588 (6)	
C11	0.22801 (16)	0.04272 (7)	1.1346 (3)	0.0669 (6)	
H11A	0.2417	0.0301	1.0502	0.080*	
C12	0.28461 (18)	0.03901 (9)	1.2443 (3)	0.0771 (8)	
H12A	0.3361	0.0241	1.2337	0.093*	
C13	0.2651 (2)	0.05718 (9)	1.3688 (3)	0.0822 (8)	
H13A	0.3033	0.0547	1.4431	0.099*	
C14	0.18916 (19)	0.07916 (10)	1.3845 (3)	0.0829 (8)	
H14A	0.1758	0.0913	1.4697	0.099*	
C15	0.13291 (17)	0.08318 (8)	1.2745 (3)	0.0703 (7)	
H15A	0.0820	0.0984	1.2860	0.084*	
N1	0.04738 (12)	0.04246 (5)	0.6758 (2)	0.0584 (5)	

### Atomic displacement parameters (Å^2^)

**Table:**

	*U*^11^	*U*^22^	*U*^33^	*U*^12^	*U*^13^	*U*^23^
C1	0.0562 (11)	0.0430 (9)	0.0576 (14)	0.0011 (8)	0.0025 (11)	0.0017 (9)
C2	0.0626 (11)	0.0412 (10)	0.0584 (14)	0.0000 (9)	0.0012 (11)	0.0017 (9)
C3	0.0792 (15)	0.0510 (11)	0.0622 (15)	0.0019 (11)	−0.0095 (13)	−0.0006 (11)
C4	0.0809 (15)	0.0638 (13)	0.0725 (16)	0.0109 (12)	−0.0124 (14)	0.0089 (12)
C5	0.0807 (15)	0.0509 (11)	0.0770 (18)	0.0140 (11)	0.0009 (15)	0.0072 (12)
C6	0.0714 (13)	0.0432 (9)	0.0681 (15)	0.0043 (9)	0.0024 (13)	−0.0018 (10)
C7	0.0649 (13)	0.0496 (11)	0.0648 (16)	−0.0036 (10)	−0.0008 (13)	0.0007 (11)
C8	0.0707 (14)	0.0538 (11)	0.0641 (16)	0.0005 (10)	−0.0006 (12)	−0.0019 (12)
C9	0.0645 (13)	0.0638 (13)	0.0627 (16)	0.0013 (11)	0.0043 (13)	−0.0040 (12)
C10	0.0632 (13)	0.0539 (11)	0.0593 (14)	−0.0054 (10)	0.0053 (12)	−0.0010 (10)
C11	0.0663 (14)	0.0743 (14)	0.0600 (16)	0.0005 (11)	0.0086 (12)	0.0008 (12)
C12	0.0688 (15)	0.0864 (18)	0.076 (2)	0.0019 (13)	0.0035 (14)	0.0137 (15)
C13	0.0814 (17)	0.0908 (19)	0.074 (2)	−0.0112 (15)	−0.0109 (17)	0.0062 (16)
C14	0.100 (2)	0.0861 (17)	0.0621 (18)	−0.0064 (16)	−0.0005 (17)	−0.0145 (15)
C15	0.0763 (15)	0.0673 (13)	0.0673 (17)	0.0006 (12)	0.0044 (14)	−0.0112 (13)
N1	0.0707 (11)	0.0445 (8)	0.0599 (13)	0.0019 (8)	−0.0044 (10)	−0.0029 (9)

### Geometric parameters (Å, °)

**Table:**

C1—C2	1.399 (3)	C8—C9	1.327 (4)
C1—C6	1.400 (3)	C8—H8A	0.9300
C1—N1	1.410 (3)	C9—C10	1.461 (3)
C2—C3	1.389 (3)	C9—H9A	0.9300
C2—C2^i^	1.485 (4)	C10—C15	1.383 (4)
C3—C4	1.390 (3)	C10—C11	1.393 (3)
C3—H3A	0.9300	C11—C12	1.375 (4)
C4—C5	1.368 (4)	C11—H11A	0.9300
C4—H4A	0.9300	C12—C13	1.362 (4)
C5—C6	1.373 (4)	C12—H12A	0.9300
C5—H5A	0.9300	C13—C14	1.373 (4)
C6—H6A	0.9300	C13—H13A	0.9300
C7—N1	1.273 (3)	C14—C15	1.375 (4)
C7—C8	1.438 (4)	C14—H14A	0.9300
C7—H7A	0.9300	C15—H15A	0.9300
			
C2—C1—C6	119.1 (2)	C7—C8—H8A	117.8
C2—C1—N1	118.93 (17)	C8—C9—C10	126.6 (2)
C6—C1—N1	121.7 (2)	C8—C9—H9A	116.7
C3—C2—C1	118.76 (18)	C10—C9—H9A	116.7
C3—C2—C2^i^	118.52 (15)	C15—C10—C11	117.3 (2)
C1—C2—C2^i^	122.67 (16)	C15—C10—C9	120.3 (2)
C2—C3—C4	121.4 (2)	C11—C10—C9	122.4 (2)
C2—C3—H3A	119.3	C12—C11—C10	121.5 (3)
C4—C3—H3A	119.3	C12—C11—H11A	119.3
C5—C4—C3	119.3 (3)	C10—C11—H11A	119.3
C5—C4—H4A	120.4	C13—C12—C11	119.8 (3)
C3—C4—H4A	120.4	C13—C12—H12A	120.1
C4—C5—C6	120.6 (2)	C11—C12—H12A	120.1
C4—C5—H5A	119.7	C12—C13—C14	120.1 (3)
C6—C5—H5A	119.7	C12—C13—H13A	120.0
C5—C6—C1	120.8 (2)	C14—C13—H13A	120.0
C5—C6—H6A	119.6	C13—C14—C15	120.1 (3)
C1—C6—H6A	119.6	C13—C14—H14A	119.9
N1—C7—C8	121.5 (2)	C15—C14—H14A	119.9
N1—C7—H7A	119.3	C14—C15—C10	121.2 (2)
C8—C7—H7A	119.3	C14—C15—H15A	119.4
C9—C8—C7	124.4 (2)	C10—C15—H15A	119.4
C9—C8—H8A	117.8	C7—N1—C1	120.32 (19)
			
C6—C1—C2—C3	−1.9 (3)	C8—C9—C10—C15	−179.7 (2)
N1—C1—C2—C3	−175.9 (2)	C8—C9—C10—C11	−2.0 (4)
C6—C1—C2—C2^i^	175.3 (2)	C15—C10—C11—C12	−0.2 (4)
N1—C1—C2—C2^i^	1.4 (3)	C9—C10—C11—C12	−177.9 (2)
C1—C2—C3—C4	0.7 (3)	C10—C11—C12—C13	0.4 (4)
C2^i^—C2—C3—C4	−176.6 (2)	C11—C12—C13—C14	0.0 (5)
C2—C3—C4—C5	0.8 (4)	C12—C13—C14—C15	−0.5 (5)
C3—C4—C5—C6	−1.1 (4)	C13—C14—C15—C10	0.7 (5)
C4—C5—C6—C1	−0.1 (4)	C11—C10—C15—C14	−0.4 (4)
C2—C1—C6—C5	1.7 (3)	C9—C10—C15—C14	177.4 (2)
N1—C1—C6—C5	175.4 (2)	C8—C7—N1—C1	−174.1 (2)
N1—C7—C8—C9	176.2 (2)	C2—C1—N1—C7	−147.5 (2)
C7—C8—C9—C10	−179.2 (2)	C6—C1—N1—C7	38.7 (3)

Symmetry codes: (i) −*x*, −*y*, *z*.
